# A P300-based cognitive assessment battery

**DOI:** 10.1002/brb3.336

**Published:** 2015-04-23

**Authors:** Aaron Kirschner, Damian Cruse, Srivas Chennu, Adrian M Owen, Adam Hampshire

**Affiliations:** 1 The Brain and Mind Institute, University of Western OntarioLondon, Ontario, Canada; 2 Department of Clinical Neurosciences, The University of CambridgeCambridge, UK; 3 The Computational, Cognitive and Clinical Neuroimaging Laboratory, Division of Brain Sciences, Imperial College LondonLondon, UK

**Keywords:** Attention, cognitive assessment, EEG, P300, reasoning, working memory

## Abstract

**Background:**

It is well established that some patients who are diagnosed as being in a vegetative state or a minimally conscious state show reliable signs of volition that may only be detected by measuring neural responses. A pertinent question is whether these patients are capable of higher cognitive processes.

**Methods:**

Here, we develop a series of EEG paradigms that probe several core aspects of cognition at the bedside without the need for motor responses and explore the sensitivity of this approach in a group of healthy controls.

**Results:**

Using analysis of ERPs alone, this method can determine with high reliability whether individual participants are able to attend a stimulus stream, maintain items in working memory, or solve complex grammatical reasoning problems.

**Conclusion:**

We suggest that this approach could form the basis of a brain-based battery for assessing higher cognition in patients with severe motor impairments or disorders of consciousness.

## Introduction

Improvements in intensive care have resulted in a surge of survivors of severe brain injury (Owen 2008). Though many survivors show significant recovery, for others, recovery is incomplete, resulting in prolonged disruption of consciousness. Neurological disorders that involve a persistent impairment of the patient's awareness of their self and environment are collectively referred to as disorders of consciousness (DOC) and include coma, vegetative state (VS), and minimally conscious state (MCS) (Owen 2008). These three conditions can be conceptualized as varying within the dimensions of wakefulness and awareness. Coma involves the cessation of both wakefulness and awareness, whereas MCS and VS patients maintain wakefulness with typical circadian rhythms of increased activity (Cruse et al. 2013). VS patients are conceived as lacking awareness, whereas MCS patients display some signs of awareness in the form of occasional purposeful behaviors. DOC misdiagnosis rates as high as 43% have been reported, typically misdiagnosing MCS as VS, with important implications for patient prognosis and medical management (Andrews et al. 1996; Kuehlmeyer et al. 2012).

In an effort to standardize DOC diagnosis, the JFK Coma Recovery Scale-Revised has emerged as a diagnostic tool with good inter-rater reliability and prognostic utility. The scale evaluates 23 operationally defined behaviors that assess auditory, visual, motor, oromotor, communication, and arousal functions (Kalmar and Giacino 2005). Despite the success of the scale, the accuracy of diagnosis in DOC remains contentious. Furthermore, it has been argued that an additional subset of patients may be misdiagnosed due to impairments in motor function, calling into question the exclusive reliance upon behavioral examination in DOC assessment (Andrews et al. 1996).

In response to these diagnostic challenges, research has been conducted into examining neural activity to diagnose DOC and understand the underlying pathophysiology. Studies of resting state activity have shown that increased functional connectivity correlates with behavioral diagnosis of patients and predicts recovery of awareness (Vanhaudenhuyse et al. 2010). Likewise, spectral patterns in EEG activity have been shown to predict patient outcome (Demertzi et al. 2008), and studies utilizing novel TMS-EEG perturbation techniques indicate that increases in long-range information transfer track the re-emergence of awareness (Rosanova et al. 2012).

Another line of research has studied neural response to sensory stimulation in DOC patients. The presence of a mismatch-negativity EEG component, indicating neural detection of deviant stimuli, has been demonstrated in DOC patients and shown to coincide with the return of functional communication (Kane et al. 1993; Fischer et al. 1999, 2010; Kotchoubey et al. 2003; Wijnen et al. 2007; Qin et al. 2008). The P300 EEG component, elicited to infrequent stimuli as well as stimuli relevant to a particular participant or task, has likewise been demonstrated in DOC populations and often tracks patient recovery (Kotchoubey et al. 2001; Cavinato et al. 2009; Faugeras et al. 2012). Furthermore, P300 amplitudes during active stimulus counting bear a significant relationship with behavioral recovery scores (Risetti et al. 2013). A hierarchical language paradigm contrasted fMRI responses to low-level auditory stimuli, intelligible speech, and semantically ambiguous sentences (Coleman et al. 2007). Localized neural responses were found in several patients in areas with demonstrated involvement in processing corresponding stimuli classes. Importantly, the level of processing demonstrated tended to correspond with subsequent recovery. These and other studies have demonstrated the potential of utilizing neural measures in characterizing residual function and recovery trajectories in DOC patients.

In contrast to the paradigms outlined above that passively present stimuli to patients, several studies have used paradigms that require the patient to actively carry out a cognitive process in response to experimenter instructions. Neural activity indicative of that process is then measured, and if established, suggests volitional behavior in the absence of an overt motor response. Owen et al. (2006) were the first to apply an active fMRI paradigm that required a patient diagnosed as VS to engage in motor or spatial imagery. Appropriate loci were shown to be more active during the appropriate imagery condition, challenging her clinical diagnosis and indicating that she could understand verbal instructions and respond appropriately. In a large-scale follow up fMRI study with 54 patients, five patients demonstrated volitional neural activity, suggesting that a significant minority of patients may be inappropriately diagnosed owing to deficits in motor output (Monti et al. 2010). Similarly, an active EEG-based paradigm demonstrated that three of 16 patients diagnosed as VS exhibited different EEG patterns in response to verbal instruction (Cruse et al. 2011).

A recent fMRI study attempted to assess executive function using a similar reasoning task (Hampshire et al. 2012). At the beginning of each trial, a patient was given a verbal reasoning problem to solve with two possible answers. The patient indicated their response by imagining either a face or a house depending on their answer. In trials where house was the correct answer, there was significantly greater activation in areas specific to spatial processing, indicating that the patient had imagined a house when it corresponded to the correct answer and was able to solve a significant number of reasoning problems correctly. This study provided the first evidence to our knowledge of successful reasoning in a patient diagnosed as MCS.

The current study sought to build on this previous work by developing and testing in controls a battery of tasks that can be used to assess higher cognitive functions without requiring an overt motor response. Due to the motor limitations of DOC patients and practical challenges of scanning patients with fMRI, the paradigm was designed to assess higher cognition using the P300b EEG response (Guger et al. 2003) that could be applied at the bedside. Three tasks were chosen for the battery: a basic command following paradigm (Kübler et al. 2001), a modified Sternberg memory span task (Sternberg 1966), and a verbal reasoning task (Münte et al. 1998; Hampshire et al. 2013). These tasks were chosen based on recent studies that highlight memory and reasoning as fundamental but dissociable components of higher cognition (Hampshire et al. 2012).

The main hypothesis of the experiment was that the battery would be able to detect command following in the first task as well as correct performance in the executive function tasks at the group and single-participant level using the P300b, which is elicited to rare, salient and attended task relevant stimuli. It was also hypothesized that the battery would be capable of determining individual differences in performance using the P300b. Lastly, exploratory analyses were conducted examining the sensitivity of the paradigm to detect correct performance as a function of testing time and participant accuracy.

## Methods

### Participants

This study was approved by The University of Western Ontario Ethics Review Board. 16 healthy adults (eight females, age: 21.1 ± 2.2 years) were recruited from the University of Western Ontario in London, Canada. All participants were right handed with no history of neurological impairment. They had good hearing and normal or corrected to normal eyesight. Two participants were excluded from analyses due to excessive movement artifacts.

### Stimuli

Recordings of eight monosyllabic English nouns were used as P300 stimuli. Words were chosen to have differing onset consonants and were matched for frequency, number of syllables, and imagability using the MRC Psycholinguistics Database (Wilson 1988).

### Task1

**Figure 1:**
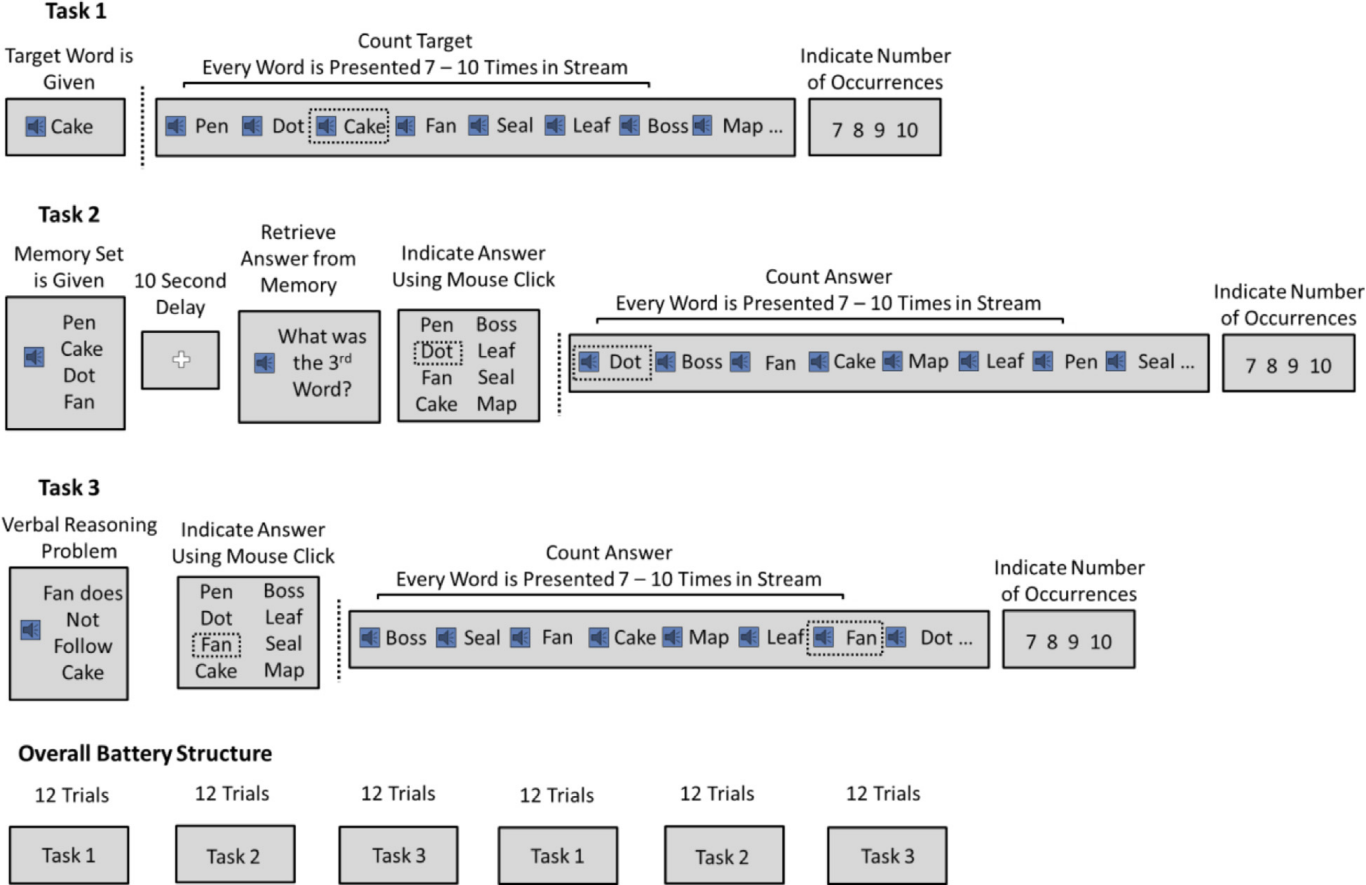
Organization of experiment and tasks.

We first implemented a basic command following paradigm using the P300b ERP. During Task 1, subsequently referred to as auditory attention (AT), participants were given a target word at the beginning of each trial. A sequence of word stimuli was subsequently played (referred to here as the “stream”), including the target word as well as all seven nontarget words. The participant's task was to internally count the number of occurrences of the target word while ignoring nontarget words (Fig.1). Counting was used because in the past it has been shown to elicit a strong P300 component, which can later be used to infer the attended word in the stream. Subjects were instructed to maintain fixation on a cross, centered on the screen during the presentation of the stream.

The stream consisted of an equal number of occurrences of all eight word stimuli, played 7–10 times each, so that the total stream length was either 56, 64, 72, or 80 words. Each stimulus was 400 msec in length, with a 100 msec between stimuli. At the end of each stream, participants indicated how many times the target word occurred. Four buttons appeared on the screen, with the choices 7, 8, 9, and 10. The participant clicked the button corresponding to their response. The purpose of the behavioral response was to test whether the participant was performing the counting task, to gauge normal performance ranges, and to provide behavioral data for cross validation of the EEG analyses. A 10 sec rest delay was given before the start of the next trial. Each block of the auditory attention task consisted of 12 trials. There were two blocks in the experiment, resulting in 24 trials total. Each word stimulus was a target three times.

### Task2

We then used the P300b to assess working memory (Fig.1). During task 2, here referred to as working memory (WM), participants were given a memory set at the beginning of each trial, consisting of 4, 6, or 8 word stimuli. Subsequently, there was a delay period of 10 sec. Following the delay, the auditory phrase “what was the xth word” was played, where x could be any ordinal position from one to the length of the memory set (e.g., 1st, 2nd, 3rd, etc.). Participants indicated their answer by clicking the appropriate button with a mouse. As per task 1 (AT), the purpose of the behavioral response was to gauge normal performance ranges and provide a benchmark to test the validity of analyzing participants' performance using the P300b ERP.

After indicating their answer, a stream of word stimuli was played, arranged as described in task 1, with 7–10 repetitions of each word stimulus. Participants counted the word stimulus corresponding to their answer and indicated its number of occurrences after the completion of the stream. Each WM block contained 12 trials with two blocks in the experiment for a total of 24 WM trials. Each memory set size was used in eight trials.

### Task3

The purpose of task 3, referred to as auditory reasoning (AR), was to assess reasoning using the P300b. Participants were given an auditory reasoning problem at the beginning of each trial (e.g., “The cake follows the pen”). The task of the participant was to determine the word in 1st position as described by the sentence (Table 1 & Fig.1). This verbal reasoning task was based on Alan Baddeley's 3 min grammatical test of IQ (Baddeley, 1968) and has been used in the past to measure reasoning abilities, as it requires the participant to maintain the sentence in mind and manipulate it according to logical rules in order to arrive at a solution (Hampshire et al. 2012). Twenty-four unique sentences were generated, one for each AR trial. Sentences were manipulated according to the verb used (precede vs. follow), negation (positive vs. negative) and whether the sentence was active or passive (“follows” vs. “is followed by”). In total, eight sentence types were generated and each was played three times over the course of the experiment. Participants indicated their answer by clicking the corresponding button using the computer mouse. The purpose of the behavioral response was again to provide a benchmark to test the validity of analyzing participants' performance using the P300b. Participants were given an unlimited amount of time to solve each problem. A 2 sec delay followed the response, followed by the auditory phrase “count that word.” A stream of word stimuli was played arranged exactly as described in task 1, with 7–10 occurrences of each word stimulus. Participants counted the word stimulus corresponding to their answer and indicated its number of occurrences after the completion of the stream with the computer mouse. Each AR block contained 12 trials, and two blocks were run in the experiment, resulting in a total of 24 AR trials.

**Table 1:** Sentence types used in AR task

Example	Precede/Follow	Active/Passive	Negative/Positive	Correct Answer
Cake precedes dot	Precede	Active	Positive	Cake
Cake does not precede dot	Precede	Active	Negative	Dot
Cake is preceded by dot	Precede	Passive	Positive	Dot
Cake is not preceded by dot	Precede	Passive	Negative	Cake
Cake follows dot	Follow	Active	Positive	Dot
Cake does not follow dot	Follow	Active	Negative	Cake
Cake is followed by dot	Follow	Passive	Positive	Cake
Cake it not followed by dot	Follow	Passive	Negative	Dot

AR, Auditory reasoning

### Assessment protocol

The experiment consisted of six blocks total with two blocks of each task (Fig.1). Each block contained 12 trials, for a total of 72 trials. The arrangement of the experiment was task 1, task 2, task 3, task 1, task 2, task 3, for all participants. The arrangement was not counterbalanced across participants as it is common practice to run neuropsychological tests in a fixed sequence to reduce factors that can confound interindividual variability measures (Tombaugh and McIntyre 1992; Fray et al. 1996). Participants were given as much time as needed to rest between blocks. The total experiment time was typically 1 h and 15 min, plus the time required for setup of the EEG recording system.

### Behavioral analysis

To test for systematic fluctuations of attention over the course of the task, the accuracy of counting target stimuli (as given by the experimenter in task 1, as indicated by participant in task 2 and 3) was compared across blocks. To test for systematic fluctuations of attention between tasks, the accuracy of counting target stimuli (as given by the experimenter in task 1, as indicated by participant in task 2 and 3) was compared across task types. The effect of set size on recall accuracy was examined in the WM task. All comparisons were performed using repeated-measures ANOVA.

To assess the effect of sentence type on performance in the AR task, a three-way repeated-measures ANOVA was conducted. Verb type (precedes vs. follows), negation (negative vs. positive sentences) and form (active vs. passive) were used as factors, each with two levels.

### EEG recording

EEG recording was performed using a G.Tec amplifier and G.Tec gel-based active electrode system (G.Tec Medical Engineering, GMBH). Electrodes were placed using the 10-10 convention and recorded from locations FC3, C3, CP3, FCZ, CZ, CPZ, FC4, C4, CP4, T7, T8, PZ, POZ, OZ, P7, P8. Data were analog filtered with a passband of 0.1–100 Hz and a notch filter at 60 Hz to reduce interference. Sampling was performed at 256 Hz, with impedances kept below 5k Ω. Scalp voltages were referenced to the right earlobe.

### EEG preprocessing

All EEG processing was performed using Matlab with EEGLAB and FieldTrip toolboxes (Delorme and Makeig 2004; Oostenveld et al. 2010). EEG was digitally filtered from 0.5 to 10 Hz with these parameters selected based on previous p300 BCI research (Guo et al. 2010). Eye and muscle artifacts were rejected using independent component analysis (ICA) (Delorme and Makeig 2004). Components that were likely the result of movement, blink, and saccade artifacts were rejected using a previously validated method utilizing kurtosis, extreme value thresholding, data improbability, and linear trending (Delorme, et al., 2010). Remaining independent components were back- projected to electrodes.

ERPs were generated by dividing trials into epochs from −200 to 1000 msec relative to word stimuli onsets. ERPs were baseline corrected by subtracting the average prestimulus magnitude from the epoch.

### Cluster mass permutation test

For the following ERP analyses, a cluster mass permutation test (CMPT) was used to test for systematic differences between conditions using a large amount of information from the EEG data while avoiding the multiple comparisons problem. This approach was first developed for fMRI (Bullmore et al. 1999) and has since been adapted for analysis of MEG and EEG data (Maris and Oostenveld 2007). For the particulars of the statistical analysis, see Appendix^1996^.

### Group-level analysis

For analysis of the AT task, ERPs were averaged within participants for all attended word stimuli and separately averaged for all unattended word stimuli, generating 28 ERPs total including two for each participant. CMPT group-level analysis was conducted in order to test for significant differences between conditions.

For analysis of the WM task, ERPs were averaged, as described above, for all correct word stimuli and separately averaged for all incorrect word stimuli within each participant. For example, in a particular WM trial, if “dot” was the correct answer, the ERP responses to “dot” would be added to the correct condition regardless of whether it was also the stimuli that the participant attended to, whereas ERP responses to all other word stimuli were added to the incorrect condition. The logic of this approach is that, should a participant solve problems significantly above chance, they will attend to the correct word stimulus and P300b ERPs will accumulate in the correct condition, whereas non-P300b ERPs (of mean magnitude zero) will accumulate in the incorrect condition, leading to a significant difference between the two conditions. However, should a participant solve problems at chance, P300b ERPs will be assigned with equal probability to both correct and incorrect bins, leading to a null result.

In the AR task, ERPs to correct word stimuli were compared to ERPs to the word stimuli that formed the other possible answer within the sentence. For example, if the sentence used was “cake precedes dot,” ERPs to the “cake” word stimuli were added to the correct stimuli condition, whereas ERP responses to “dot” were added to the incorrect condition. This approach was used because a participant could potentially listen for both words in each trial. If all stimuli other than the correct word stimuli were added to the incorrect bin, even though one of the incorrect stimuli ERPs contained a P300b it would be diluted by the other incorrect stimuli and be significantly lower in magnitude, giving rise to a positive result when the participant did not solve the problem correctly. Adding only the ERP to the incorrect stimuli in the sentence to the incorrect condition avoids this issue.

### Single-participant level EEG analysis

For analysis of the AT task at the single-participant level, ERPs to individual attended word stimuli were compared to ERPs to the unattended word stimuli. For analysis of the WM task, ERPs to correct word stimuli were compared to ERPs to incorrect word stimuli. For analysis of the AR task, ERPs to correct word stimuli were compared to ERPs to only the incorrect word stimuli used in that same sentence.

### Prediction of individual differences from P300b responses

The purpose of the individual differences analysis was to explore whether the magnitude of P300b responses predicted performance as indicated by behavioral measures. As discussed below, participants performed largely at a ceiling level with no significant differences between conditions in the AR task. Consequently, differences between participants were predicted from ERPs only in the WM task.

The theory motivating this analysis is that, should a participant perform at 100%, their ERP magnitude to correct word stimuli would be equal to their ERP magnitude to attended stimuli, as all correct stimuli would also be attended stimuli. Conversely, if the participant performed at 50%, only half of the correct word stimuli would also be attended. Given that half of the ERPs in the correct condition will be P300b ERPs, whereas half will be non-P300b ERPs of average magnitude zero, the correct ERP will be half as large as the attended ERP magnitude. If a performance coefficient is calculated between zero and one, the resulting correct ERP magnitude should be the attended ERP magnitude multiplied by this coefficient. For example, if the attended P300b magnitude is 4чv for a participant who performs at 75%, the correct P300b magnitude should be 3чv.

In order to test this predictive model, ERP magnitudes were first calculated within each participant at each WM difficulty level (four, six, or eight item memory sets), resulting in three ERPs for each participant. Time × electrode values that were in the spatiotemporal regions selected by the CMPT were averaged to calculate a mean magnitude within each of the three conditions for each participant. This magnitude was then divided by the average ERP magnitude to attended stimuli from the AT task to calculate normalized ERP magnitude (NM). A NM was calculated for each condition in each participant, resulting in 42 NM total. Each NM magnitude had a paired performance score calculated from behavioral data.

The unique and combined relationship between set size, NM and performance was analyzed using a generalized linear model with memory set size as a factor and NM as a covariate. The purpose of this test was to first examine whether NM could be used to predict individual differences in performance overall. The analysis also modeled the prediction of performance from NM within difficulty levels (by factoring out the effects of difficulty level on performance). Second, this test was able to analyze whether interactions existed between set size and NM such that NM was more predictive of performance depending on the level of difficulty. Following this analysis, a correlation test was performed between NM and performance within each difficulty level to examine the nature of the linear relationship at each level.

Due to the larger differences in variance of participant performance in the hardest memory sets with eight items (discussed below), an additional single-participant level analysis CMPT was conducted with only four and six item memory set trials included.

### Relationship between P300b significance, time, and performance

As discussed above, the normalized magnitude of the P300b response to correct stimuli should vary linearly with performance. Therefore, the *P*-value for differences between ERP responses to correct versus incorrect word stimuli should also vary with performance such that better performance decreases the *P*-value and increases statistical confidence that the participant is able to perform the task. Similarly, as task time increases, the number of stimuli in each condition likewise increases, also decreasing the *P*-value and adding to statistical confidence. Furthermore, these two variables are related. Better performance decreases the amount of time required to attain a significant *P*-value, whereas worse performance increases the time required to achieve the same *P*-value. The nature of this relationship is crucial to the purposes of this paradigm, as these parameters determine the sensitivity of the test to detect accurate performance, or lack thereof, as well as the length of time required for the battery to reach a significant level of confidence.

The relationship between task time, performance and *P*-value in the overall task was modeled using a Monte Carlo simulation. The following Monte Carlo procedure was used:

1. Within each participant, ERP responses to stimuli that were actually attended were collected by selecting ERP responses to word stimuli that corresponded to the participants' behavioral response in each trial.
2. A random selection of *n* (where n increases with time on task) attended ERPs were selected from the attended ERP set, whereas 7*n ERP responses were randomly selected from the unattended ERP set.
3. A single-participant level CMPT was performed between these two sets to attain a *P*-value.
4. For each value of *n*, steps two and three were repeated 100 times to decrease the effect of particular selections on the resultant *P*-value.
5. These 100 *P*-values were averaged to attain mean *P*-value at that n for the given participant.
6. The value of *n* was increased in multiples of 25 to simulate increasing time, with steps 2–5 repeated at each value of *n*.

The effect of performance was simulated by inserting an intervening step between steps 2 and 3. Attended and unattended ERPs were swapped between conditions depending on simulated performance. For example, if the simulated performance was 0.6, 40% of the attended ERPs were randomly swapped for an equal number of unattended ERPs between conditions. Performance levels of 0.3 to 1 were used in increments of 0.1.

## Results

### Behavioral results

The accuracy of counting in the AT task was 70% across all levels, with no significant differences between blocks (*F*(5, 65) = 1.985, *P* = 0.092) or level.

On the WM task, participants averaged 80% across all three set sizes, with 97% correct for four item sets, 80% correct for six item sets and 64% for eight item sets. All participants scored above chance at all difficulty levels. There was a significant effect of memory set size on accuracy of recall, *F*(2, 26) = 22.701, *P* < 0.001. Individual comparisons of accuracy between memory set sizes also revealed significant differences, with four item sets recalled significantly better than six items (*P* < 0.001) and eight items (*P* < 0.001), and six items sets remembered significantly better than eight items (*P* = 0.005).

On the AR task, participants averaged 95% correct across all sentence types. All but one participant scored above chance at all levels. There was no significant main effect of the verb used (precedes vs. follows), *F*(1, 13) = −0.11, *P* > 0.05, negation, *F*(1, 13) = 0.51, *P* > 0.05, or passive vs. active sentences, *F*(1, 13) = 0.21, *P* > 0.05. There were no significant 2 or 3-way interactions). The absence of significant differences in this task indicates ceiling performance.

### EEG results—group level analysis

**Figure 2:**
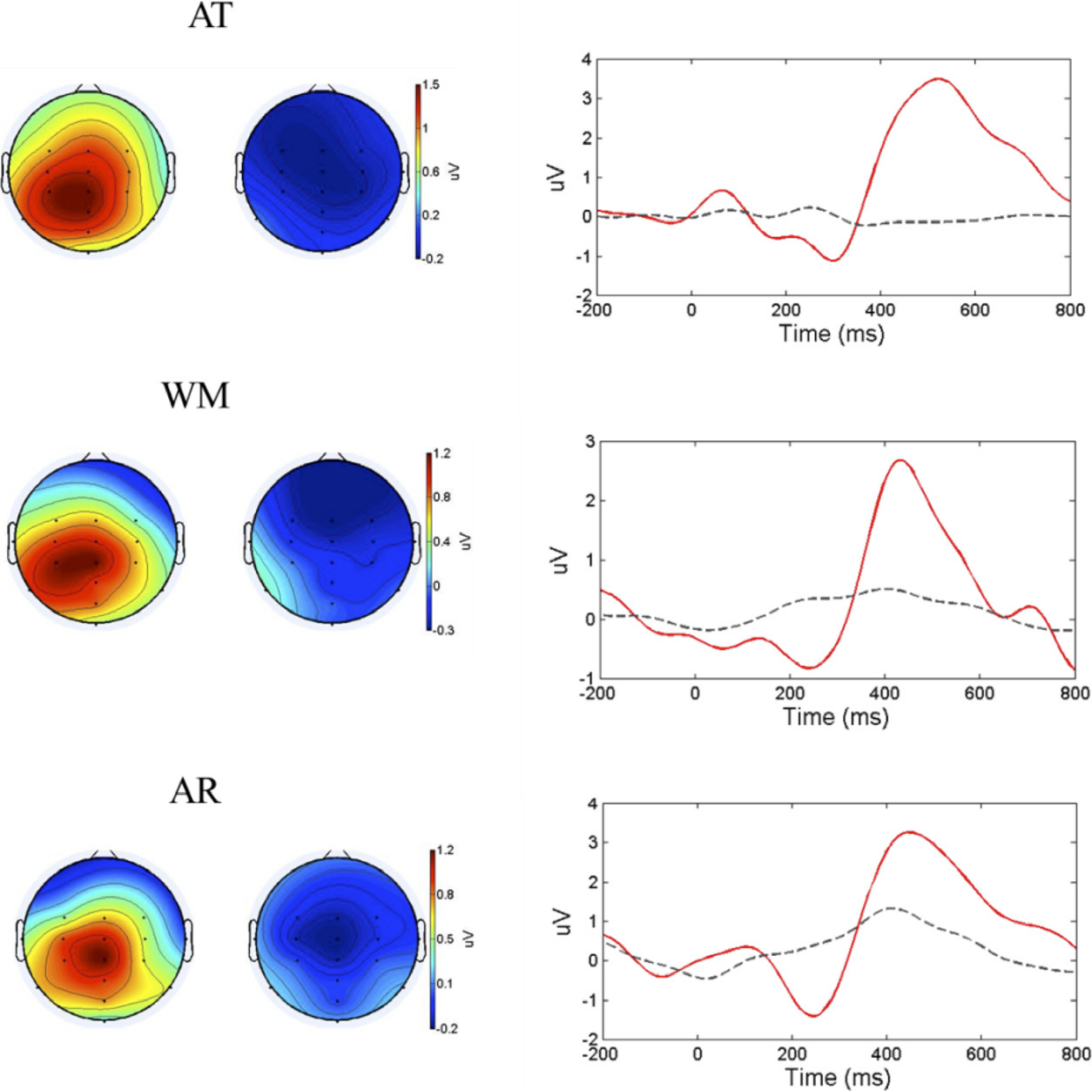
Group average scalp maps in all three tasks at 450 msec post stimulus onset. Attended (AT) and correct (WM and AR) topomaps on left side, unattended (AT) and incorrect (WM and AR) ERPs on right. ERP time courses for attended and correct (red line) versus unattended and incorrect (black line) over electrode CPz.

At the group level, the CMPT revealed that ERPs were significantly larger to attended word stimuli than unattended word stimuli in the AT task (*P* < 0.001). CMPT also revealed significantly larger ERPs to correct word stimuli compared to incorrect word stimuli in both the WM task (*P* = 0.002) and the AR task (*P* = 0.003). Group-averaged topomaps showed that for all tasks the P300b response was most prominent in posterior electrodes (Fig.2).

### Single-participant level

**Figure 3:**
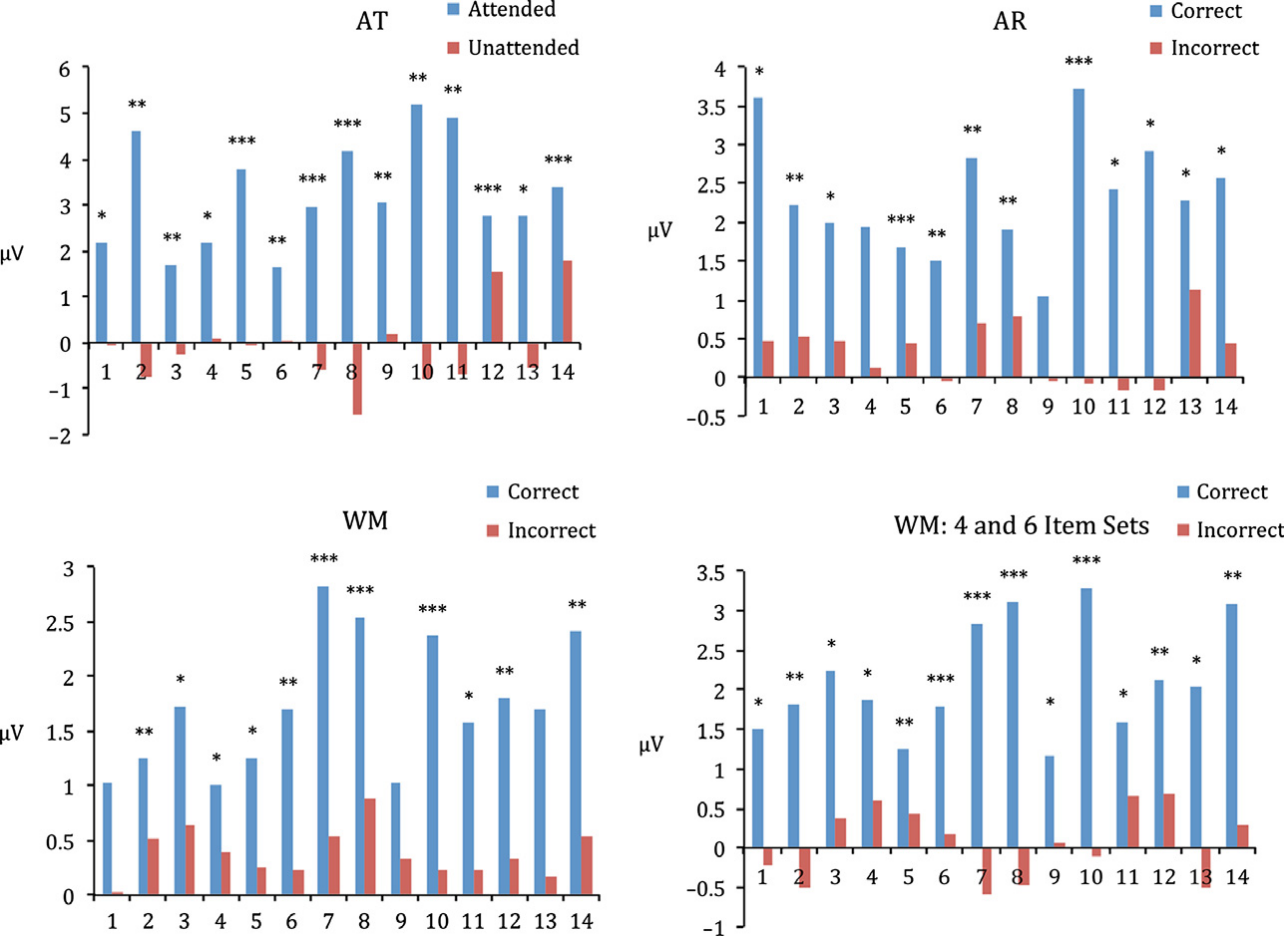
Top left: Mean voltage magnitude within largest CMPT cluster for each participant in AT task. Bottom left: Mean voltage magnitude within largest CMPT cluster for each participant in WM task. Top right: Mean voltage magnitude within largest CMPT cluster for each participant in AR task. Bottom Right: Mean voltage magnitude within largest CMPT cluster for each participant in WM task with eight item sets excluded. ***P < 0.001, **P < 0.01, *P < 0.05.

CMPT at the single-participant level for the AT task revealed a significant difference between ERPs to attended stimuli versus unattended word stimuli for all participants, a significant difference between ERPs to correct versus incorrect word stimuli in 11/14 participants in the WM task, and a significant difference between ERPs to correct versus incorrect word stimuli in 13/14 participants in the AR task (Fig.3).

Due to increased variance of participant performance in the eight item memory sets, the attended word stimuli often did not correspond to the correct word stimuli. The CMPT analysis was not significant for three participants, which may have been caused by the inclusion of incorrect trials from the eight item set. In order to explore this possibility, a second CMPT analysis was conducted with the inclusion of word stimuli from only four and six item memory sets. The restricted CMPT revealed a significant difference between ERPs to correct and incorrect word stimuli in all 14 participants.

### Prediction of individual differences from P300b components

**Figure 4:**
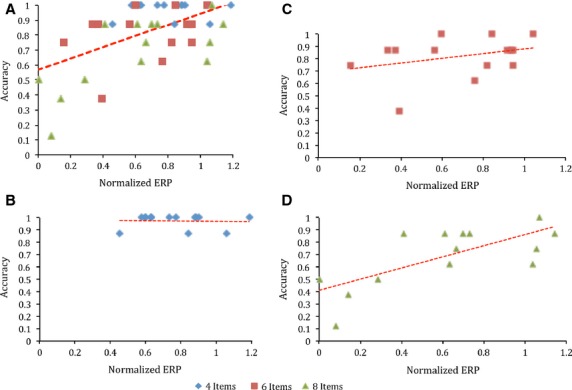
(A) Relationship between accuracy within memory set sizes and normalized ERP magnitude. Each data point represents the overall accuracy of a single participant in a single WM set size condition. (B) Relationship between accuracy within four item memory sets and normalized ERP magnitude. (C) Relationship between accuracy within six item memory sets and normalized ERP magnitude. (D) Relationship between accuracy within eight item memory sets and normalized ERP magnitude. Each point represents data from one participant. Red line represents line of best fit.

Results from the generalized linear model demonstrated that memory set size significantly predicted performance, χ^2^(2, *n* = 42) = 15.123, *P* < 0.001, with performance decreasing as memory set size increased. Importantly, normalized ERP magnitude (NM) predicted performance even when the general effect of set size was factored out, demonstrating that NM predicted participant differences within individual memory set sizes; χ^2^(1, *n* = 42) = 6.742, *P* = 0.009 (Fig.4). The generalized linear model also revealed a significant interaction between memory set size and normalized ERP magnitude, χ^2^(2, *n* = 42) = 6.149, *P* = 0.049, suggesting that the predictive power of ERP magnitude was modulated according to memory set size.

In order to explain the linear relationship between NM and performance within each memory set size, three correlation tests were performed, one at each memory set size. NM was not significantly correlated with accuracy within four item sets (Fig.4), *r*(12) = −0.44, *P* > 0.05 or six item sets, *r*(12) = 0.321, *P* > 0.05. NM and accuracy were significantly correlated within the eight item set size, *r*(12) = 0.712, *P* = 0.002.

### Significance as a function of number of targets

**Figure 5:**
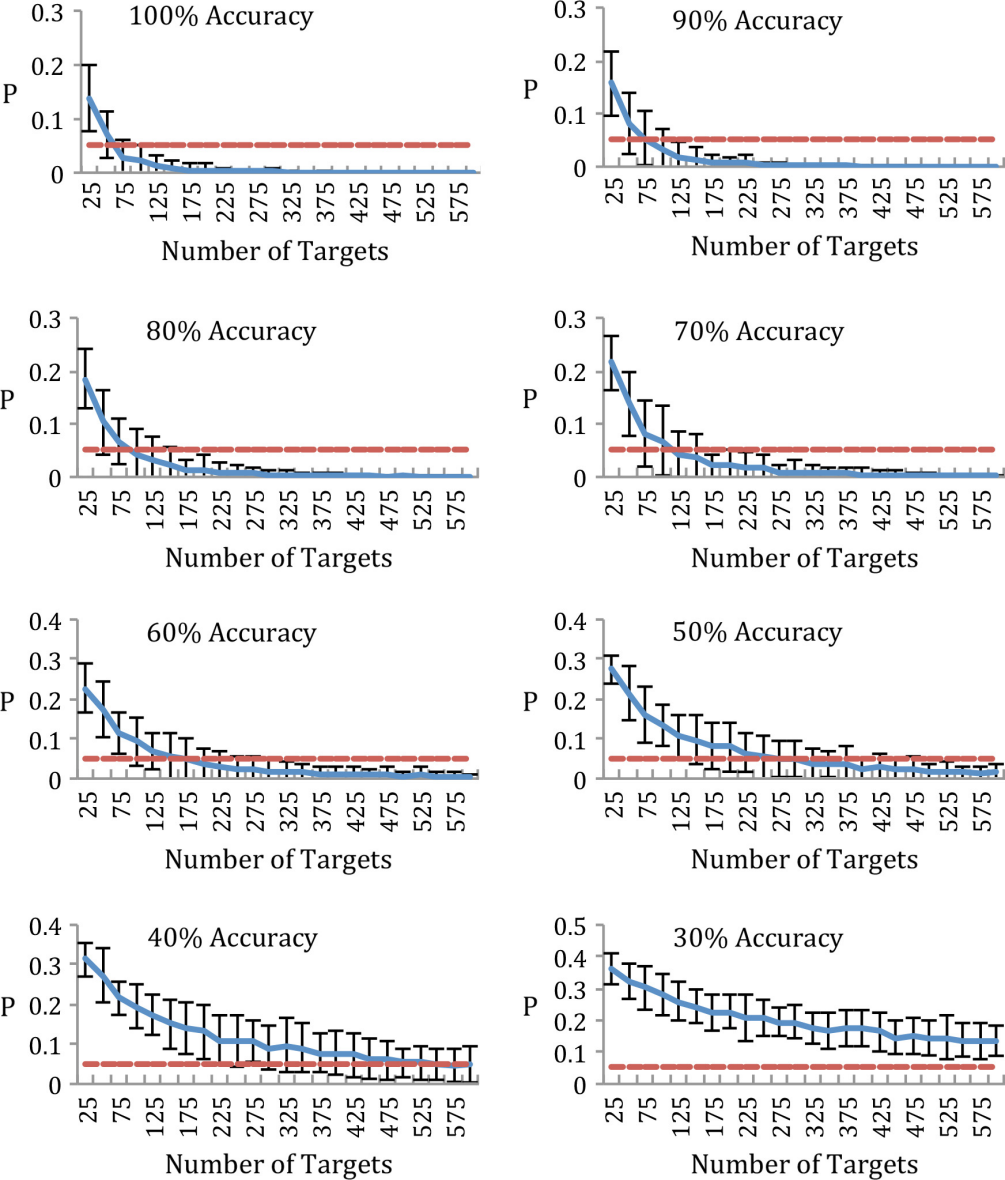
Mean CMPT *P*-value for all participants as a function of number of targets. Delivery of 25 targets (one target per eight stimuli, 500 msec per stimulus, 200 total stimuli) takes approximately 1 min and 40 sec. Error bars represent standard error. Red dotted line represents *P* = 0.05.

An exploratory analysis was conducted to investigate the combined effects of task duration and subject accuracy on the statistical sensitivity of the test (Fig.5). At 100% simulated accuracy, mean *P*-value was less than 0.05 after 75 attended stimuli, with large variation between participants. As the number of stimuli increased, *P*-value decreased asymptotically to zero while variance likewise decreased. Given that each attended stimuli plus interstimulus interval is 500 msec and it always accompanies seven unattended stimuli of the same duration, the average amount of time per attended stimuli is approximately 4 sec. Seventy-five attended stimuli therefore take approximately 5 min to deliver, not counting the time taken to pose questions within trials. As accuracy decreased, a larger number of stimuli were required to reach the same level of significance. At 90% accuracy, 100 attended stimuli were required to achieve the same *P*-value. At 60% accuracy, 175 attended stimuli were required, whereas at 30% accuracy a *P*-value of less than 0.05 was unattainable with 600 attended stimuli. In principle, because chance performance was 12.5%, any accuracy above this threshold should be detectible with an arbitrarily large number of stimuli. However, factors such as participant fatigue and changes in electrode placement and impedance place an upper limit on the number of stimuli that can be delivered.

## Discussion

Overall, the battery was largely successful and has the potential to be used for directly assessing executive function in DOC patients. At the group level, both the WM and AR tasks generated a significant P300b to correct word stimuli, demonstrating that overall performance across participants was significantly above chance. The single-participant level results were the most promising. A significant P300b was found in 11/14 participants in the WM task and 13/14 participants in the AR task. When eight item memory sets were removed from analysis, P300b responses from all 14 participants were significant in the WM task. The adjusted battery was therefore able to detect correct performance in tasks requiring cognitive processes in 27/28 cases using the P300b ERP.

In the AT task, a significant P300b response was found in all participants analyzed, demonstrating that the P300b has the potential to be used as a command following paradigm for detecting residual awareness in DOC patients (Dias et al. 2007), and replicating previous studies that successfully utilized the auditory P300b to operate a BCI system (Klobassa et al. 2009; Kübler et al. 2009).

This approach could be used as a complementary means of detecting volition in addition to motor imagery paradigms developed previously (Chennu et al. 2013). In standard behavioral DOC assessment batteries, a variety of motor output channels are used to examine command following to rule out the possibility that damage to specific channels obscures the patient's ability to follow instructions (Kalmar and Giacino 2005). An analogous approach should be taken when using active paradigms that employ volitional modulations in neural activity. Damage to the motor system might prevent the patient from performing motor imagery. Likewise, damage to top down attention systems could prevent the patient from attending to target stimuli. By utilizing several command following paradigms to target a broad set of neural systems, a larger proportion of patients can be adequately assessed.

The second hypothesis of the study was that the battery would be able to predict individual differences in performance using the P300b response and results here were somewhat mixed. In the AR task, ceiling performance precluded meaningful variation in performance to predict. That is, the task can detect whether an individual is able to reason, but at these difficulty levels, cannot differentiate between high and low reasoning abilities in controls. In the WM task, there was a significant relationship between ERP magnitude and accuracy. This result was largely driven by the variance in the eight item memory sets, however, and ceiling performance in four item and six item memory sets again reduced individual differences. Within the eight item memory set, there was a reasonably high correlation, suggesting that more difficult tasks that increase variability should result in higher predictive accuracy, though further research is required in order to better confirm this hypothesis.

In general, the ability to detect core cognitive functions is much more important than characterizing normative performance in patients, at least at the outset. As DOC patients are presumed to have at most minimal levels of consciousness, demonstration of higher cognitive functions would profoundly challenge a patient's diagnosis. Near ceiling performance is ideal for attaining this result in a timely and robust manner, as demonstrated by the Monte Carlo simulation. In the AR task as well as the smaller WM set sizes, this difference was significant for almost all participants.

However, once the presence of these functions is established, providing a more fine-grained analysis of the patient's particular capacities is instrumental in determining the suitable amount and complexity of information to present, allowing an appropriate level of patient autonomy, and facilitating comparisons across patients. Unlike detection of above-chance performance, significant divergence in participant performance is necessary to assess individual differences.

Unfortunately, the particular conditions that maximize the likelihood of optimal detection versus assessment are in tension in the current paradigm. High performance is required for detection, whereas variability in performance across conditions is necessary for assessment. In order to accommodate both of these motivations, a modified paradigm is recommended for future exploration. Rather than using a randomly presented, predefined number of trials at each difficulty level, working memory and reasoning problems should be presented in a sequence of increasing difficulty. Furthermore, the presentation of problems should be controlled dynamically in concert with real-time statistical analysis of ERPs. At the beginning of the neuropsychological battery, problems at the lowest level of difficulty should be presented first while ERPs to correct versus incorrect word stimuli are compared online as data is collected. Once the statistical difference between conditions reaches a predefined threshold, the patient can proceed to a higher level of difficulty, with more challenging problems presented. Likewise, the statistical power for determining a lack of difference can be calculated in real-time and given a similar threshold for determining that the patient cannot perform at that level. Similar to other dynamic neuropsychological tests, the last difficulty level at which a patient can perform satisfactorily can be taken as their capacity.

This approach has several advantages. Normalizing ERPs is not required for estimating performance, eliminating the need to establish baseline ERP responses during each testing session. Likewise, because difficulty is increased as soon as significance is reached, extraneous time need not be spent establishing performance at lower levels. Lastly, this procedure would better accommodate individual differences in ERP discernibility. As shown in Fig.3, participants varied in performance and by extension the number of trials they required for a significant result. Using a set number of trials therefore expends unnecessary time with some participants, while failing to detect a valid difference in others. Likewise, as demonstrated in the Monte Carlo simulation, a patient performing at 70–90%, though still acceptable, may require addition trials to reach significance. Given the variability in EEG activity and patient characteristics, a testing paradigm that adapts to the patient should be adopted, both in the present battery as well as future active neuroimaging assessment paradigms.

This study developed and evaluated a battery of neuropsychological tests that can be administered to behaviorally unresponsive patients using the P300b ERP component. In the majority of participants (93%), the ability to perform tasks requiring cognitive functions was detected without the need to rely on motor output. The magnitude of the P300b component was related to individual differences in performance, but only with sufficient variability between participants. Using Monte Carlo simulations, it was demonstrated that the battery could detect significant performance with a mean time of 5 min, with the potential to be shortened with better optimization. As communication with DOC patients using BCIs becomes widespread, it will become increasingly necessary to assess residual cognitive function for both ethical and scientific purposes. As part of a larger battery of neuropsychological tests, the approach developed here has the potential to provide a standardized assessment tool for clinicians and scientists.

## Conflict of Interest

None declared.

## Appendix

For the following ERP analyses, a cluster mass permutation test (CMPT) was used, adapted from Cruse et al. (2012). The general motivation behind this procedure is the recognition that EEG recordings generate a large number of time samples within each ERP, with this number multiplied by the number of electrodes. It would require a substantial number of comparisons to compare each time-electrode sample between the two conditions, each increasing the probability of type 1 errors. Due to the need to correct for multiple comparisons, the sensitivity of the test is severely diminished. Instead, the cluster mass approach provides a test statistic that is based on clustering adjacent spatial-temporal samples. This approach was first developed for fMRI (Bullmore et al. 1999) and has since been adapted for analysis of MEG and EEG data (Maris and Oostenveld 2007). In order to generate the test statistic, the following procedure is used:

1. For every time sample in a predefined window at every electrode, compare the EEG signal between the two conditions.
  For each participant, there will be n trials from condition one and m trials from condition two. Each trial is a matrix of time × electrode EEG voltage samples. Therefore, for each time-electrode point, there will be n samples from condition one and m samples from condition two. Perform an independent samples *t*-test for each time-electrode point in the matrix between the two conditions. A temporal analysis window of 300 to 800 msec post stimulus onset was used following based on previous P300b research (Guo et al. 2010).
2. Select all time-electrode points whose *P*-value is lower than a predefined threshold. In this study, *P* < 0.05 was used, following conventional practice (Maris and Oostenveld 2007).
3. Cluster significant points that are both spatially and temporally adjacent. Points must be temporally adjacent by immediately following one another and spatially adjacent by virtue of being recorded from neighboring electrodes
4. For each cluster, sum all *t*-values of significant time-electrode points.
5. Select the cluster with largest summed *t*-values. This sum forms the test statistic.

From this analysis, a single value is generated, referred to subsequently as the cluster mass value (CMV). In order to perform statistical analyses on the differences between conditions, a nonparametric permutation approach is taken. For comparisons between conditions at the single-participant level, the following procedure is used:

1. Collect all trials of the two experimental conditions in a single set. Each trial includes the time-varying voltage recorded at all electrodes.
2. Randomly draw as many trials from this combined dataset as there are trials in condition one. Place those trials into subset one. Place the remaining trials in subset two. This results in a random partition.
3. Calculate the CMV on this random partition.
4. Repeat steps 2 and 3 10,000 times. This large number of permutations allows a more precise characterization of the probability distribution and dilutes the effects of statistical anomalies.
5. Place the test statistic that was actually observed into the histogram created in step 4.
6. Calculate the proportion of random partitions that resulted in a larger CMV than the observed one to derive a *P*-value.

A similar approach is used for CMPT group-level analysis. In order to generate the test statistic, the following procedure is used:

1. For each participant, an average ERP for each condition is calculated. Each participant is given a single matrix of time × electrode values for each condition. Time windows were restricted to 300–800 msec post stimulus onset similar to single-participant level analysis.
2. Each condition consists of a set of time × electrode matrices, one for each participant. Conduct a paired-samples *t*-test at each time-electrode point to determine points that differ significantly between the two conditions.
3. Cluster significant points (*P* < 0.05) that are both spatially and temporally adjacent. Points must be temporally adjacent by preceding or following one another and spatially adjacent by virtue of being recorded from neighboring electrodes
4. For each cluster, sum all *t*-values.
5. Take the largest of the cluster-level statistics.

From this analysis, a CMV is generated. In order to perform statistical analyses on the differences between conditions, a nonparametric permutation approach is used, albeit differing slightly from the previous method:

1. Within individual participants, permute the average ERPs in each condition. For example, within participant one, the value of the average ERP from condition one is reassigned to condition two, and vice versa. The participants for which this exchange takes place are selected randomly
2. Calculate the CMV on this permuted data set.
3. Repeat steps 1 and 2 10,000 times. This number of permutations allows a more precise characterization of the probability distribution and dilutes the effects of statistical anomalies.
4. Place the test statistic that was actually observed into the histogram created in step 3.
5. Calculate the proportion of random partitions that resulted in a larger CMV than the observed one to derive a *P*-value.

In both group level and single-participant level CMPTs, a *P*-value less than a predefined alpha level (*P* < 0.05 in the current study) resulted in a rejection of the null hypothesis. As mentioned above, this test is useful because a single statistical test is conducted for each comparison between conditions, controlling for the multiple comparisons problem. In contrast to parametric approaches, this approach does not make assumptions about the distribution of the test statistic. Importantly, CMPT allows spatiotemporal localization of significant changes in electrophysiological activity in a data-driven manner.
